# Environmental pesticide exposure and non-Hodgkin lymphoma survival: a population-based study

**DOI:** 10.1186/s12916-022-02348-7

**Published:** 2022-04-26

**Authors:** Christina Poh, John D. McPherson, Joseph Tuscano, Qian Li, Arti Parikh-Patel, Christoph F. A. Vogel, Myles Cockburn, Theresa Keegan

**Affiliations:** 1 Division of Medical Oncology, University of Washington, Seattle, WA USA; 2 Fred Hutchinson Cancer Center, Seattle, WA USA; 3 Division of Hematology/Oncology, UC Davis Comprehensive Cancer Center, Sacramento, CA USA; 4 Department of Biochemistry and Molecular Medicine, UC Davis, Sacramento, CA USA; 5 Veterans Administration, Northern California Healthcare System, Sacramento, CA USA; 6 California Cancer Reporting and Epidemiologic Surveillance Program, UC Davis, Sacramento, CA USA; 7 Department of Environmental Toxicology and the Center for Health and the Environment, UC Davis, Davis, CA USA; 8 Department of Preventive Medicine, Keck School of Medicine, University of Southern California, Los Angeles, CA USA

**Keywords:** Pesticide, Lymphoma, Survival

## Abstract

**Background:**

There is evidence indicating that pesticide exposure is a risk factor for non-Hodgkin lymphoma (NHL) development. However, the association between pesticide exposure and NHL survival is not well-established.

**Methods:**

Using the California Cancer Registry, we identified patients with a first primary diagnosis of NHL from 2010 to 2016 and linked these patients with CalEnviroScreen 3.0 to obtain production agriculture pesticide exposure to 70 chemicals from the state-mandated Pesticide Use Reporting (PUR) by census tract from 2012 to 2014. In addition, data from PUR was integrated into a geographic information system that employs land-use data to estimate cumulative exposure to specific pesticides previously associated with NHL (glyphosate, organophosphorus, carbamate, phenoxyherbicide, and 2,4-dimethylamine salt) between 10 years prior up to 1 year after NHL diagnosis. Multivariable Cox proportional hazards regression models were used to evaluate the association between total pesticide exposure from CalEnviroScreen 3.0 and individual pesticide exposure from geographic land use data and lymphoma-specific and overall survival.

**Results:**

Among 35,808 NHL patients identified, 44.2% were exposed to pesticide in their census tract of residence. Glyphosate, organophosphorus, carbamate, phenoxyherbicide, and 2,4-dimethylamine salt exposure was observed in 34.1%, 26.0%, 10.6%, 14.0%, and 12.8% of NHL patients, respectively. Total pesticide exposure at the time of diagnosis was not associated with lymphoma-specific or overall survival. In addition, no association was consistently found between glyphosate, organophosphorus, carbamate, phenoxyherbicide, and 2,4 dimethylamine salt exposure and lymphoma-specific or overall survival.

**Conclusions:**

Although we found no consistent associations between agricultural pesticide exposure at the neighborhood level and worse survival, these results provide a platform for designing future studies to determine the association between pesticide and NHL.

**Supplementary Information:**

The online version contains supplementary material available at 10.1186/s12916-022-02348-7.

## Background

Non-Hodgkin lymphoma (NHL), a diverse group of malignant neoplasms variously derived from the clonal expansion of B, T, or natural killer cells, is the most common hematologic malignancy, constituting 4.3% of new neoplasms in the United States and 3.3% of all cancer deaths [1]. It is generally associated with a favorable prognosis, with a 5-year relative survival of 73.2% [1]. While the etiology of most NHLs continues to be largely unknown, its pathogenesis likely represents a complex process involving the accumulation of multiple genetic mutations, together with underlying immune dysfunction [2].

Pesticide includes a diverse group of chemicals or biologic agents applied in agricultural, industrial/commercial, and residential settings to control pests [3, 4]. In the United States alone in 2012, over 1.1 billion pounds of pesticide was utilized [5]. Despite its widespread usage, pesticide exposure has been associated with multiple adverse health effects, including cancer development [6].

There is evidence indicating that pesticide exposure is a risk factor for NHL development. Occupational agricultural exposure has been associated with a higher incidence rate of NHL in multiple studies [7–12] and the International Agency for Research on Cancer determined exposure to three commonly used agricultural organophosphates (glyphosate, malathion, and diazinon) to be carcinogenic and associated with NHL development in 2015 [13]. Genotoxicity and reactive oxygen species generation in healthy cells by pesticide used for agricultural production is likely involved in the transformation of healthy lymphocytes into clonal ones. Prolonged environmental exposure to pesticide could lead to clonal expansion of cells with specific tumorigenic alterations and immunodeficiency, potentially contributing to pathogenesis and resistance [14–16]. Chronic exposure could also lead to adaptive mechanisms for DNA repair and antioxidant activity, undermining chemotherapy regimens [17]. Furthermore, there is a possibility that carcinogenic effects might not result from direct mutagenic or genotoxic mechanisms from the pesticide itself. For example, endocrine disruption or an indirect initiation of inflammation must be considered as tumor-promoting mechanisms of pesticide [18].

While emphasis has been placed on evaluating pesticide as a causative agent for NHL development, investigation on its association with NHL prognosis also has the potential to provide important insight into NHL etiology and pathogenesis. Only one study thus far has investigated the association between pesticide exposure and NHL treatment outcomes. In a French multicenter retrospective cohort study of 244 patients with diffuse large B-cell lymphoma (DLBCL), occupational exposure to pesticide was found to be associated with increased treatment failure and poorer event-free and overall survival compared to those not exposed to pesticide [19]. These findings have yet to be validated and suggest additional adverse implications of pesticide exposure, rationalizing further study. Therefore, we identified NHL patients from the California Cancer Registry and linked these individuals with the statewide pesticide use reporting database to determine the association of pesticide exposure with lymphoma-specific and overall survival.

## Methods

### Databases

The California Cancer Registry (CCR) is the statewide population-based cancer surveillance system which maintains records about all malignancies diagnosed in California, with the exception of basal and squamous cell carcinoma of the skin. The CCR estimates that >98% of cancer diagnoses in California are captured. CCR data includes the date of diagnosis, primary anatomic site, histologic type, American Joint Committee on Cancer (AJCC) stage, initial type of treatment, and sociodemographic information.

The pesticide use reporting (PUR) program contains records of all agricultural pesticide use in California since 1990 and is maintained by the California Department of Pesticide Regulation. Under this state-mandated program, all pesticide applications to parks, golf courses, cemeteries, rangeland, pastures, along roadside and railroads, and all postharvest pesticide treatments of agricultural commodities must be reported monthly. Primary exceptions to reporting requirements include residential and some industrial and institutional applications.

For this study, we utilized CalEnviroScreen 3.0, California’s science-based mapping tool developed by the Office of Environmental Health Hazard Assessment and California Environment Protection Agency which uses the PUR database to obtain scores of average pollution burden for every census tract in the state.

In addition, data from PUR was integrated with data from land-use surveys [20, 21] (based on California’s Public Land Survey System (PLSS), specifying the exact location of crops on which pesticide was most likely used) to estimate cumulative exposure to specific pesticides. Specifically, the locations of agricultural pesticide applications were reported according to the PLSS, a grid that parcels land into sections with an area of approximately one square mile and is used in the 30 westernmost states formed from lands in the public domain. To improve the scale of available estimates, we combined this data with information from California land-use surveys, the countywide, large-scale surveys (1,24,000, or 1 inch = 2000 feet) of land use and crop cover conducted every 7–10 years. These data are available electronically with land-use types existing as contiguous polygons that are individually linked to their respective attribute information (e.g., land use type, acreage) in a database table. The reconciliation of these datasets was completed using geospatial software that allows for the generation of point estimates (and estimates of their variation) across a continuous spatial surface using the combined datasets. For each location (e.g., a residential address) we derived a summation of pesticide use from the surrounding area.

### Patient cohort

Using the CCR, we identified patients with a first primary diagnosis of NHL from 1/1/2010 to 12/31/2016 using specific International Classification of Diseases-Oncology, 3rd edition (ICD-O-3) codes [22–24]. Patients with missing data, including date of diagnosis or date of follow-up (*n*=462), without a known cause of death (*n*=322) and those with human immunodeficiency virus infection (HIV)/acquired immune deficiency syndrome (AIDS) (*n*=1338) or a second malignancy (*n*=3083) after NHL diagnosis were excluded.

### Estimating pesticide exposure

Identified NHL patients were merged by census tract for CalEnviroScreen 3.0 pesticide data. Production agricultural pesticide exposure to 70 chemicals was obtained from 2012 to 2014; the total pounds of selected active ingredients for each census tract was divided by each census tract area to obtain total pounds (lbs) per square mile, averaged over the 3 years [25].

In addition, NHL patients were merged with data from PUR and land-use surveys to estimate for cumulative exposure to specific pesticides previously associated with increased NHL incidence, glyphosate [26, 27], organophosphorus [28–30], carbamate [12, 31], phenoxyherbicide [32–34], and 2,4-dimethylamine salt [30, 35]. Cumulative pesticide exposure was measured as lbs of pesticide applied per acre/month within 2000 meters from residence at diagnosis, between 10 years prior up to 1 year after NHL diagnosis to include a significant amount of lead time prior to diagnosis through treatment of NHL. Pesticide exposure was then categorically grouped into levels of low, mid, and high exposure based on the tertile distribution of exposure levels for each pesticide.

### Covariates

From the CCR, we obtained patient demographics which include gender, race/ethnicity, age and stage at diagnosis, modality of initial therapy, health insurance status, neighborhood socioeconomic status (SES), and rural or urban medical service study area (MSSA) at diagnosis. Initial treatment was identified as either chemotherapy and/or radiation therapy. In addition, patients were broadly categorized into the NHL subtypes of DLBCL, follicular lymphoma, Burkitt lymphoma, mantle cell lymphoma, marginal zone lymphoma, small lymphocytic lymphoma, lymphoblastic lymphoma, other B-cell lymphomas, T/NK-cell neoplasms, and unspecified lymphomas.

### Statistical analyses

Multivariable Cox proportional hazards regression models were used to identify the association between pesticide exposure and lymphoma-specific and overall survival. Models considered five common types of pesticide (glyphosate, organophosphorus, carbamate, phenoxyherbicide, and 2,4-dimethylamine salt) and NHL subtype separately. Models included variables with a priori reasons for inclusion based on previous studies: gender, race/ethnicity, age and stage at diagnosis, modality of initial therapy, health insurance status, neighborhood SES and rural or urban MSSA [36–38]. We also examined whether associations between pesticide exposure and survival differed by race/ethnicity in multivariable Cox models performed separately in Hispanic/Latino, non-Hispanic white, Asian/Pacific Islander, and African American patients.

Lymphoma-specific survival was measured from the date of diagnosis to the date of death from lymphoma whereas overall survival considered death from all causes. Patients who died from causes other than lymphoma were censored at the time of death in the analysis of lymphoma-specific survival. Patients alive at the study end date (12/31/2016) were censored at this time or at the date of last known contact. Median follow-up time was calculated using the reverse Kaplan-Meier method [39, 40].

For all regression analyses, the proportional hazard assumption was assessed using Schoenfeld residuals [41]. Variables, including the stage at diagnosis and modality of initial treatment, that violated the proportional hazard assumption were included as stratification variables. Survival analyses were done using SAS 9.4 and all statistical tests were two-sided; a *P*-value of less than 0.05 was considered statistically significant. This study was approved by the University of California, Davis Institutional Review Board.

## Results

We identified 35,808 patients between 2010 and 2016 with a first primary diagnosis of NHL who met our criteria. Baseline demographics are shown in Table 1. Overall, 44.2% of all NHL patients were exposed to pesticide in their census tract of residence. Pesticide exposure was higher in Hispanic/Latino (46.5%) and non-Hispanic white (45.6%) than Asian/Pacific Islander (37.2%) and African American (34.9%) patients with NHL. Almost half (46.8%, 45.8%, 45.4%, 44.4%, and 42.2%) of NHL patients with Burkitt lymphoma, follicular lymphoma, small lymphocytic lymphoma, DLBCL, and T/NK-cell neoplasms had pesticide exposure, respectively.

**Table 1:** Baseline characteristics of non-Hodgkin lymphoma patients, California, 2010–2016

Characteristics	NHL patients		
	Total	Pesticide exposure^a^	No pesticide exposure
	*N* = 35,808 (%)	*N* = 15,834 (%)	*N* = 19,974 (%)
Gender			
Male	19,401 (54.2)	8529 (53.9)	10,872 (54.4)
Female	16,399 (45.8)	7301 (46.1)	9098 (45.5)
Race/ethnicity			
Non-Hispanic White	20,079 (56.1)	9163 (57.9)	10,916 (54.7)
African American	1629 (4.5)	568 (3.6)	1061 (5.3)
Hispanic/Latino	8601 (24.0)	4003 (25.3)	4598 (23.0)
Asian/Pacific Islander	4494 (12.6)	1673 (10.6)	2821 (14.1)
Other/unknown	1005 (2.8)	427 (2.7)	578 (2.9)
Age at diagnosis			
0–44	4711 (13.2)	1960 (12.4)	2751 (13.8)
45–64	12,836 (35.9)	5669 (35.8)	7167 (35.9)
65–84	15,194 (42.4)	6834 (43.2)	8360 (41.9)
85+	3067 (8.6)	1371 (8.7)	1696 (8.5)
NHL histology			
DLBCL	13,980 (39.0)	6203 (39.2)	7777 (38.9)
Follicular lymphoma	6384 (17.8)	2921 (18.4)	3463 (17.3)
Burkitt lymphoma	515 (1.4)	241 (1.5)	274 (1.4)
Mantle cell lymphoma	1528 (4.3)	695 (4.4)	833 (4.2)
Marginal zone lymphoma	3582 (10.0)	1538 (9.7)	2044 (10.2)
Small lymphocytic lymphoma	1400 (3.9)	636 (4.0)	764 (3.8)
Other B-cell lymphoma	960 (2.7)	389 (2.5)	571 (2.9)
T/NK-cell lymphoid neoplasms	3246 (9.1)	1369 (8.6)	1877 (9.4)
Lymphoblastic lymphoma	443 (1.2)	192 (1.2)	251 (1.3)
Unspecified	3770 (10.5)	1650 (10.4)	2120 (10.6)
Stage at diagnosis			
I/II—limited	15,205 (42.5)	6681 (42.2)	8524 (42.7)
III/IV—advanced	17,976 (50.2)	7975 (50.4)	10,001 (50.1)
Modality of initial therapy			
Chemotherapy	20,501 (57.3)	9094 (57.4)	11,407 (57.1)
Radiation	5168 (14.4)	2324 (14.7)	2844 (14.2)
Health insurance status			
Private	17,061 (47.6)	7401 (46.7)	9660 (48.4)
Public/none	17,105 (47.8)	7702 (48.6)	9403 (47.1)
Unknown	1642 (4.6)	731 (4.6)	911 (4.6)
Neighborhood socioeconomics status (quintile)			
1 (lowest)	5305 (14.8)	2345 (14.8)	2960 (14.8)
2	6408 (17.9)	2954 (18.7)	3454 (17.3)
3	7354 (20.5)	3421 (21.6)	3933 (19.7)
4	8080 (22.6)	3647 (23.0)	4433 (22.2)
5 (highest)	8661 (24.2)	3467 (21.9)	5194 (26.0)
Area of residence			
Rural	4849 (13.5)	3270 (20.7)	1579 (7.9)
Urban	30,959 (86.5)	12,564 (79.3)	18,395 (92.1)
Pesticide exposure			
Glyphosate		12,195 (34.1)	
Organophosphorus		9310 (26.0)	
Carbamate		3801 (10.6)	
Phenoxyherbicide		5005 (14.0)	
2,4-Dimethylamine salt		4593 (12.8)	
Composite (70 chemicals)		14,056 (39.5)	

^a^Cumulative pesticide exposure was measured as pounds of pesticide applied per acre/month within 2000 m from residence at diagnosis, between 10 years prior up to 1 year after NHL diagnosis

*DLBCL* diffuse large B-cell lymphoma

Forty-four percent of patients with both limited and advanced NHL stage had pesticide exposure. Forty percent of NHL patients in the highest SES quintile had pesticide exposure compared to 44.2% in the lowest SES quintile. Pesticide exposure was found among 67.4% of NHL patients in rural and 40.9% in urban MSSA. Glyphosate, organophosphorus, carbamate, phenoxyherbicide, and 2,4-dimethylamine salt exposure were observed in 34.1%, 26.0%, 10.6%, 14.0%, and 12.8% of patients, respectively.

Median follow-up time was 2.99 years (95% CI: 2.95–3.04) for all patients with a primary diagnosis of NHL. Using CalEnviroScreen 3.0 data, we demonstrated no association between total pesticide exposure and worse lymphoma-specific survival (HR: 0.97; 95% CI: 0.92–1.02) or overall survival (HR: 0.97; 95% CI: 0.93–1.02). In addition, pesticide exposure was not associated with worse lymphoma-specific and overall survival when stratified by NHL subtypes (Table 2) or race/ethnicity (data not shown in Tables).

**Table 2:** Association between pesticide exposure* (versus no exposure) and lymphoma-specific and overall survival among non-Hodgkin lymphoma patients overall and by histologic subtype

Histologic subtype	*N*	Percentage of the population exposed^a^	Lymphoma-specific survival	Overall survival
			HR (95% CI)^b^	HR (95% CI)^b^
All NHL	35,600	39.5	0.97 (0.92, 1.02)	0.97 (0.93, 1.02)
DLBCL	13,922	39.1	0.94 (0.87, 1.01)	0.95 (0.90, 1.01)
Follicular lymphoma	6333	17.8	0.87 (0.72, 1.06)	0.89 (0.76, 1.03)
Burkitt lymphoma	513	1.4	0.91 (0.61, 1.35)	0.91 (0.63, 1.32)
Mantle cell lymphoma	1516	4.3	0.95 (0.77, 1.17)	1.03 (0.86, 1.24)
Marginal zone lymphoma	3559	10.0	1.18 (0.84, 1.65)	1.02 (0.82, 1.27)
Small lymphocytic lymphoma	1397	3.9	0.79 (0.45, 1.40)	0.90 (0.67, 1.22)
Other B-cell lymphoma	952	2.7	1.04 (0.58, 1.86)	1.07 (0.74, 1.56)
T/NK-cell lymphoid neoplasms	3219	9.0	1.05 (0.90, 1.22)	1.04 (0.90, 1.19)
Lymphoblastic lymphoma	443	1.2	0.82 (0.43, 1.60)	0.84 (0.54, 1.28)
Unspecified	3746	10.5	0.96 (0.83, 1.11)	0.95 (0.85, 1.06)

^a^Exposed patients are defined as those residing in a census tract with any pesticide exposure from 2012 to 2014

^b^Models were adjusted for gender, race/ethnicity, age at diagnosis, health insurance status, neighborhood socioeconomic status, rural or urban medical service study area and were stratified by stage at diagnosis and modality of initial therapy which included chemotherapy or radiation

*NHL* non-Hodgkin lymphoma, *DLBCL* diffuse large B-cell lymphoma

**Fig. 1:**
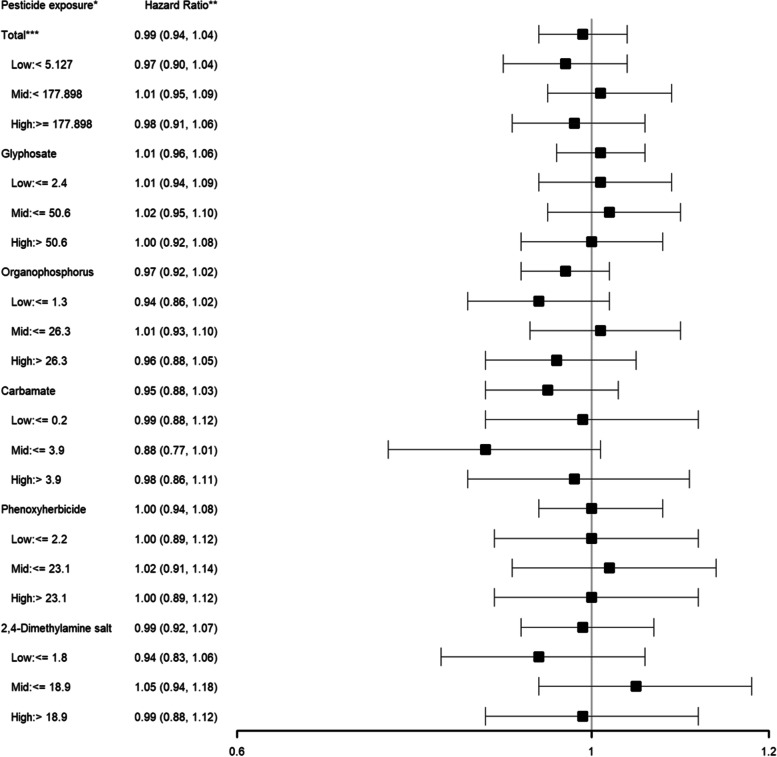
Association between varying levels of individual pesticide exposure* (versus no exposure) and lymphoma-specific survival among non-Hodgkin lymphoma patients

**Fig. 2:**
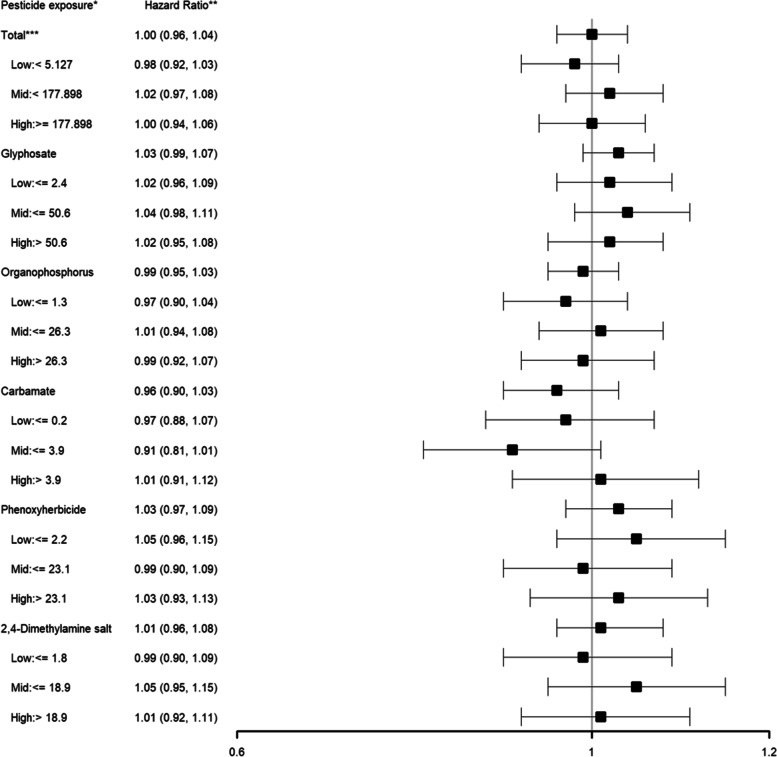
Association between varying levels of individual pesticide exposure* (versus no exposure) and overall survival among non-Hodgkin lymphoma patients

With the integration of land-use surveys which allowed for analysis of the effects of specific pesticides, glyphosate, organophosphorus, carbamate, phenoxyherbicide and 2,4-dimethylamine salt exposure was not associated with worse lymphoma-specific or overall survival (lymphoma-specific survival: glyphosate HR: 1.01; 95% CI: 0.96–1.06, organophosphorus HR: 0.97; 95% CI: 0.92–1.02, carbamate HR: 0.95; 95% CI: 0.88–1.03, phenoxyherbicide: 1.00; 95% CI: 0.94–1.08, 2,4-dimethylamine salt HR: 0.99; 95% CI: 0.92–1.07; overall survival: glyphosate HR: 1.03; 95% CI: 0.96–1.07, organophosphorus HR: 0.99; 95% CI: 0.95–1.03, carbamate HR: 0.96; 95% CI: 0.90–1.03, phenoxyherbicide: 1.03; 95% CI: 0.97–1.09, 2,4-dimethylamine salt HR: 1.01; 95% CI: 0.96–1.08) (Figs. 1 and 2). When examined by race/ethnicity, no consistent association between varying levels of pesticide exposure and lymphoma-specific or overall survival was found. Furthermore, when examined by pesticide tertile levels, all hazard ratios showed no association between varying levels of pesticide exposure and lymphoma-specific survival or overall survival.

When examined by NHL histology, most pesticide exposure was not associated with worse lymphoma-specific survival, with the exception of carbamate exposure in lymphoblastic lymphoma (HR: 3.87; 95% CI: 1.66–9.01) (Table 3). With further stratification of carbamate exposure into tertile levels among lymphoblastic lymphoma patients, mid (HR: 6.77; 95% CI: 2.20–20.80), but not low (HR: 1.37; 95% CI: 0.18–10.57) or high (HR: 4.19; 95% CI: 0.96–18.26) tertile levels were associated with worse lymphoma-specific survival (Additional file 1: Table S1). In contrast, phenoxyherbicide and 2,4-dimethylamine salt exposure was associated with superior lymphoma-specific survival in mantle cell lymphoma with hazard ratios of 0.68 (95% CI: 0.50–0.92) and 0.71 (95% CI: 0.92–1.02), respectively. The effect persisted with further stratification of phenoxyherbicide and 2,4-dimethylamine salt exposure into tertile levels among mantle cell lymphoma patients with superior lymphoma-specific survival observed with low (HR 0.51; 95% CI: 0.31–0.85), but not mid (HR 0.85; 95% CI: 0.50–1.45) or high (HR 0.77; 95% CI: 0.48–1.24) phenoxyherbicide tertiles and low (HR 0.47; 95% CI: 0.27–0.82), but not mid (HR 1.05; 95% CI: 0.62–1.78) nor high (HR 0.82; 95% CI: 0.49–1.35) 2,4-dimethylamine salt tertiles.

**Table 3:** Association between individual pesticide exposure^a^ (versus no exposure) and lymphoma-specific and overall survival among non-Hodgkin lymphoma patients, stratified by histologic subtype

Histologic subtype	*N*	Percentage of population exposed^a^	Lymphoma-specific survival	Overall survival
			HR (95% CI)^b^	HR (95% CI)^b^
Total^c^				
DLBCL	13,980	44.4	0.97 (0.91, 1.03)	0.98 (0.92, 1.03)
Follicular lymphoma	6384	45.8	0.99 (0.82, 1.18)	1.01 (0.88, 1.16)
Burkitt lymphoma	515	46.8	1.23 (0.84, 1.79)	1.31 (0.92, 1.86)
Mantle cell lymphoma	1528	45.5	0.91 (0.74, 1.11)	0.90 (0.76, 1.07)
Marginal zone lymphoma	3582	42.9	0.76 (0.54, 1.05)	0.94 (0.77, 1.16)
Small lymphocytic lymphoma	1400	45.4	1.14 (0.69, 1.88)	1.02 (0.78, 1.32)
Other B cell lymphoma	960	40.5	0.75 (0.44, 1.30)	0.89 (0.63, 1.26)
T/NK cell lymphoid neoplasms	3246	42.2	0.95 (0.82, 1.10)	0.96 (0.85, 1.10)
Lymphoblastic lymphoma	443	43.3	0.84 (0.46, 1.54)	0.88 (0.59, 1.30)
Unspecified	3770	43.8	1.03 (0.91, 1.18)	1.03 (0.93, 1.14)
Glyphosate				
DLBCL	13,980	34.4	0.98 (0.92, 1.05)	1.00 (0.94, 1.06)
Follicular lymphoma	6384	35.4	1.01 (0.84, 1.22)	1.08 (0.94, 1.25)
Burkitt lymphoma	515	38.8	1.09 (0.74, 1.61)	1.18 (0.82, 1.69)
Mantle cell lymphoma	1528	34.1	0.94 (0.77, 1.16)	0.95 (0.80, 1.14)
Marginal zone lymphoma	3582	32.4	0.98 (0.69, 1.39)	1.00 (0.80, 1.25)
Small lymphocytic lymphoma	1400	34.4	1.25 (0.75, 2.09)	0.97 (0.73, 1.27)
Other B cell lymphoma	960	30	0.96 (0.53, 1.73)	1.05 (0.72, 1.52)
T/NK cell lymphoid neoplasms	3246	32	1.02 (0.87, 1.18)	1.03 (0.90, 1.18)
Lymphoblastic lymphoma	443	33.4	1.18 (0.62, 2.25)	1.19 (0.79, 1.79)
Unspecified	3770	33.9	0.97 (0.85, 1.12)	1.03 (0.93, 1.15)
Organophosphorus				
DLBCL	13,980	26.7	0.92 (0.86, 1.00)	0.95 (0.90, 1.02)
Follicular lymphoma	6384	25.9	0.89 (0.72, 1.09)	0.94 (0.81, 1.10)
Burkitt lymphoma	515	29.7	1.21 (0.82, 1.79)	1.28 (0.89, 1.83)
Mantle cell lymphoma	1528	25.9	0.93 (0.74, 1.17)	0.92 (0.76, 1.11)
Marginal zone lymphoma	3582	24.4	0.73 (0.50, 1.09)	0.96 (0.76, 1.22)
Small lymphocytic lymphoma	1400	27.7	0.95 (0.55, 1.61)	0.98 (0.74, 1.30)
Other B-cell lymphoma	960	23.5	0.91 (0.50, 1.66)	0.93 (0.63, 1.37)
T/NK-cell lymphoid neoplasms	3246	24.8	0.99 (0.84, 1.16)	0.99 (0.86, 1.14)
Lymphoblastic lymphoma	443	25.7	0.87 (0.42, 1.80)	1.06 (0.68, 1.66)
Unspecified	3770	25.6	1.07 (0.93, 1.24)	1.04 (0.92, 1.16)
Carbamate				
DLBCL	13,980	10.8	0.90 (0.81, 1.00)	0.94 (0.86, 1.03)
Follicular lymphoma	6384	10.9	0.92 (0.69, 1.22)	0.96 (0.77, 1.19)
Burkitt lymphoma	515	8.7	1.08 (0.59, 1.97)	1.29 (0.75, 2.20)
Mantle cell lymphoma	1528	10.4	0.90 (0.66, 1.25)	0.88 (0.66, 1.16)
Marginal zone lymphoma	3582	10.5	1.12 (0.70, 1.79)	0.98 (0.72, 1.34)
Small lymphocytic lymphoma	1400	10.6	0.87 (0.42, 1.81)	0.94 (0.64, 1.40)
Other B-cell lymphoma	960	10.1	0.56 (0.20, 1.59)	0.99 (0.56, 1.75)
T/NK-cell lymphoid neoplasms	3246	10.3	0.83 (0.66, 1.05)	0.87 (0.71, 1.06)
Lymphoblastic lymphoma	443	7.7	3.86 (1.66, 9.01)	1.49 (0.76, 2.95)
Unspecified	3770	10.7	1.02 (0.83, 1.25)	0.98 (0.83, 1.15)
Phenoxyherbicide				
DLBCL	13,980	14	1.04 (0.95, 1.15)	1.07 (0.99, 1.16)
Follicular lymphoma	6384	14.5	1.00 (0.78, 1.28)	1.03 (0.85, 1.24)
Burkitt lymphoma	515	14.6	0.97 (0.58, 1.62)	1.09 (0.68, 1.75)
Mantle cell lymphoma	1528	14.3	0.68 (0.50, 0.92)	0.74 (0.57, 0.95)
Marginal zone lymphoma	3582	12.1	0.89 (0.53, 1.48)	0.79 (0.57, 1.11)
Small lymphocytic lymphoma	1400	15	1.08 (0.58, 2.02)	0.99 (0.70, 1.41)
Other B-cell lymphoma	960	11.4	1.15 (0.54, 2.43)	1.16 (0.70, 1.94)
T/NK-cell lymphoid neoplasms	3246	13.8	0.95 (0.77, 1.18)	0.94 (0.78, 1.13)
Lymphoblastic lymphoma	443	14	1.48 (0.64, 3.44)	1.39 (0.79, 2.45)
Unspecified	3770	14.7	0.91 (0.75, 1.11)	0.98 (0.84, 1.14)
2,4-Dimethylamine salt				
DLBCL	13,980	12.9	1.03 (0.93, 1.13)	1.06 (0.97, 1.15)
Follicular lymphoma	6384	13.4	1.04 (0.80, 1.35)	1.01 (0.83, 1.24)
Burkitt lymphoma	515	13.6	0.99 (0.59, 1.68)	1.14 (0.70, 1.84)
Mantle cell lymphoma	1528	12.9	0.71 (0.52, 0.98)	0.77 (0.59, 1.01)
Marginal zone lymphoma	3582	11.3	0.89 (0.53, 1.50)	0.83 (0.59, 1.17)
Small lymphocytic lymphoma	1400	14	1.15 (0.61, 2.16)	1.03 (0.72, 1.47)
Other B-cell lymphoma	960	10.3	1.15 (0.53, 2.50)	1.17 (0.69, 1.98)
T/NK-cell lymphoid neoplasms	3246	12.9	0.94 (0.75, 1.17)	0.93 (0.77, 1.13)
Lymphoblastic lymphoma	443	12.6	1.66 (0.70, 3.90)	1.19 (0.64, 2.19)
Unspecified	3770	13.1	0.86 (0.70, 1.06)	0.96 (0.82, 1.12)

^a^ Cumulative pesticide exposure was measured as pounds of pesticide applied per acre/month within 2000 m from residence at diagnosis, between 10 years prior up to 1 year after NHL diagnosis

^b^Models were adjusted for gender, race/ethnicity, age at diagnosis, health insurance status, neighborhood socioeconomic status, rural or urban medical service study area and were stratified by stage at diagnosis and modality of initial therapy which included chemotherapy or radiation

^c^Total refers to glyphosate, organophosphorus, carbamate, phenoxyherbicide, and 2,4-dimethylamine salt combined

*NHL* non-Hodgkin lymphoma, *DLBCL* diffuse large B-cell lymphoma

Pesticide exposure was not associated with worse overall survival in any of the NHL histologies examined. In contrast, phenoxyherbicide exposure was associated with superior overall survival in mantle cell lymphoma (HR 0.74; 95% CI: 0.57–0.95). With further stratification of phenoxyherbicide exposure in tertile levels among mantle cell lymphoma patients, low (HR 0.63; 95% CI: 0.43–0.94), but not mid (HR 0.77; 95% CI: 0.47–1.24) nor high tertiles (HR 0.86; 95% CI: 0.58–1.27) were also associated with superior overall survival.

## Discussion

In this large population-based study, we observed pesticide exposure to be prevalent among patients diagnosed with NHL, with high pesticide exposure particularly observed in Hispanics/Latinos and non-Hispanic whites. Using multiple measures of agricultural pesticide exposure, we did not observe pesticide exposure to be consistently associated with worse lymphoma-specific or overall survival.

Our study observed pesticide exposure among 46.5% of Hispanics/Latinos and 45.6% of non-Hispanic white NHL patients. Given the racial/ethnic distribution of hired agricultural workers in the United States (57% Hispanic/Latino and 32% non-Hispanic white), we would expect Hispanics/Latinos to have the highest exposure level. However, our neighborhood-level pesticide exposure does not capture occupational exposures outside the neighborhood or exposure to non-agricultural pesticides within the neighborhood, limiting our ability to fully capture pesticide exposure levels. Indeed, our analysis showed no consistent associations between pesticide exposure and worse lymphoma-specific and overall survival among each race/ethnicity.

Our findings are supported by an analysis of 232 NHL patients from the Danish Diet, Cancer and Health cohort which found no association between organochlorine pesticide concentrations in adipose tissue and survival [42]. In contrast, a French multicenter retrospective cohort study of 244 DLBCL patients treated with anthracycline-based immunochemotherapy and with complete occupational histories observed increased treatment failure and poorer event-free and overall survival with occupational pesticide exposure [19]. The survival disadvantage was especially notable in patients with agricultural occupations compared with other exposed patients. In addition, these outcome differences persisted even though patients without pesticide exposure were more likely to present with disseminated disease and patients with and without exposure had no differences in demographic characteristics, initial presentation, international prognostic index, and treatment protocol. Our differences in findings may relate to the type of study design or our inability to classify groups with high personal pesticide exposure; our study included an unselected population of nearly all lymphoma patients in California, whereas the French study included a much smaller sample of patients from 6 medical centers. Given potential associations between pesticide exposure and NHL prognosis, more detailed studies are warranted.

Pesticides exert their toxic effect through various mechanisms such as reactive oxidative damage, chromosomal damage, and alteration of DNA repair mechanisms. Cytogenetic analysis of peripheral blood lymphocytes in 120 Serbian people exposed to pesticide demonstrated higher levels of chromosomal aberrations when compared to those without pesticide exposure [43]. Similarly, evaluation of bone marrow samples from commercial, family, and organic farmers showed higher levels of chromosomal abnormalities in commercial and family farmers compared to organic farmers [44]. In addition, low concentrations of an organophosphorus, malathion, although not associated with cell death, was shown to upregulate oncogenes, downregulate tumor suppressor genes and induce gene expression of human B and T lymphocytes and immunoglobulin production which are involved in the initiation, progression, and pathogenesis of cancer [45]. These results highlight the need for further correlative and molecular studies, including DNA damage signature analysis and RNA sequencing, to identify biomarkers associated with pesticide exposure.

There are limitations to our study. Studies pertaining to the effects of pesticide are investigated worldwide due to its widespread use; pesticide usage patterns likely vary with different locations and the relative importance of residential pesticide exposure is limited to areas with prevalent pesticide use. Our study also utilized census tract as a proxy for pesticide exposure because of a lack of more detailed patient-reported data, such as direct vs. indirect exposure to pesticide (e.g., occupational exposure vs. exposure via aerosol or food contamination), differences in routine activity among patients in the same census tract, or duration of patient residency in a specified census tract. These factors are important in understanding the association between pesticide exposure and NHL. Furthermore, although the PUR database is regarded as one of the most extensive worldwide, guidelines for pesticide use reporting according to the California Department of Pesticide Regulation may limit its utility, as it does not capture details of residential exposure, which accounts for up to 10% of overall pesticide exposure in the United States [5]. Another limitation includes our short follow-up time (~3 years) for lymphoma-specific and overall survival which may underestimate the potential association between pesticide exposure and NHL prognosis.

Strengths of this study include the large population-based cohort of 35,808 patients with NHL which allowed for a robust multivariate analysis of outcomes. In addition, this study used pesticide usage data available from state resources which is an objective measurement of pesticide usage. Lastly, this is one of the few studies that specifically investigate the association between pesticide exposure and NHL treatment outcomes and survival, as previous studies primarily focused on the association between pesticide exposure and NHL incidence.

## Conclusions

In summary, pesticide exposure is common among patients with NHL, particularly among Hispanics/Latinos and non-Hispanic whites. Although we found no consistent associations between agricultural pesticide exposure at the neighborhood level and worse survival, additional investigations with more details of personal pesticide exposure and longer follow-up time are warranted based on potential associations between pesticide exposure and NHL prognosis.

## Supplementary Information

**Additional file 1: Table S1.** Association between individual pesticide exposure and lymphoma-specific and overall survival among non-Hodgkin lymphoma patients, stratified by histologic subtype and varying pesticide exposure levels.

## Data Availability

Available upon request.

## Abbreviations

AIDS: Acquired immune deficiency syndrome
AJCC: American Joint Committee on Cancer
CCR: California Cancer Registry
DLBCL: Diffuse large B-cell lymphoma
HIV: Human immunodeficiency virus
HR: Hazard ratio
lbs: Total pounds
MSSA: Medical service study area
NHL: Non-Hodgkin lymphoma
PUR: Pesticide Use Reporting
SES: Socioeconomic status
