# Supplementary material for: Environmental pesticide exposure and non-Hodgkin lymphoma survival: a population-based study
Source: BMC Med. 2022 Apr 26;20:165. doi: 10.1186/s12916-022-02348-7 (PMC9040269; doi:10.1186/s12916-022-02348-7)
Supplement: Supplementary file 1 — Additional file 1: Table S1. Association between individual pesticide exposure and lymphoma-specific and overall survival among non-Hodgkin lymphoma patients, stratified by histologic subtype and varying pesticide exposure levels. [file 12916_2022_2348_MOESM1_ESM.docx]

**Additional file 1: Table S1: Association between individual pesticide exposure* and lymphoma-specific and overall survival among non-Hodgkin lymphoma patients, stratified by histologic subtype and varying pesticide exposure levels**

|  | lymphoma-specific survival | | overall survival |
| --- | --- | --- | --- |
| Pesticide exposure** | HR (95% CI)*** | | HR (95% CI)*** |
| Total**** |  | |  |
| DLBCL |  | |  |
| Low:< 5.127 | 0.98 (0.89, 1.07) | | 0.97 (0.90, 1.06) |
| Mid:< 177.898 | 0.94 (0.86, 1.04) | | 0.97 (0.89, 1.05) |
| High:>= 177.898 | 0.98 (0.89, 1.08) | | 0.99 (0.91, 1.08) |
| Follicular lymphoma |  | |  |
| Low:< 5.127 | 1.13 (0.88, 1.44) | | 1.08 (0.89, 1.31) |
| Mid:< 177.898 | 0.96 (0.74, 1.24) | | 1.03 (0.85, 1.25) |
| High:>= 177.898 | 0.87 (0.67, 1.14) | | 0.91 (0.74, 1.12) |
| Burkitt lymphoma |  | |  |
| Low:< 5.127 | 1.06 (0.60, 1.87) | | 1.02 (0.59, 1.75) |
| Mid:< 177.898 | 1.43 (0.90, 2.27) | | 1.59 (1.04, 2.43) |
| High:>= 177.898 | 1.13 (0.64, 1.98) | | 1.23 (0.73, 2.08) |
| Mantle cell lymphoma |  | |  |
| Low:< 5.127 | 0.91 (0.69, 1.20) | | 0.90 (0.71, 1.15) |
| Mid:< 177.898 | 0.90 (0.68, 1.20) | | 0.89 (0.70, 1.13) |
| High:>= 177.898 | 0.90 (0.67, 1.22) | | 0.91 (0.70, 1.18) |
| Marginal zone lymphoma |  | |  |
| Low:< 5.127 | 0.69 (0.43, 1.10) | | 0.99 (0.75, 1.30) |
| Mid:< 177.898 | 0.86 (0.53, 1.39) | | 0.92 (0.67, 1.25) |
| High:>= 177.898 | 0.74 (0.44, 1.25) | | 0.91 (0.66, 1.25) |
| Small lymphocytic lymphoma |  | |  |
| Low:< 5.127 | 1.15 (0.57, 2.32) | | 1.06 (0.74, 1.54) |
| Mid:< 177.898 | 1.00 (0.48, 2.06) | | 0.76 (0.50, 1.14) |
| High:>= 177.898 | 1.28 (0.66, 2.47) | | 1.25 (0.88, 1.77) |
| Other B-cell lymphoma |  | |  |
| Low:< 5.127 | 0.63 (0.28, 1.45) | | 0.69 (0.41, 1.18) |
| Mid:< 177.898 | 0.47 (0.16, 1.36) | | 0.92 (0.54, 1.56) |
| High:>= 177.898 | 1.13 (0.55, 2.35) | | 1.13 (0.69, 1.85) |
| T/NK-cell lymphoid neoplasms |  | |  |
| Low:< 5.127 | 0.92 (0.74, 1.15) | | 1.01 (0.84, 1.21) |
| Mid:< 177.898 | 0.98 (0.80, 1.20) | | 0.94 (0.78, 1.13) |
| High:>= 177.898 | 0.95 (0.77, 1.18) | | 0.95 (0.78, 1.14) |
| Lymphoblastic lymphoma |  | |  |
| Low:< 5.127 | 0.68 (0.26, 1.73) | | 0.69 (0.35, 1.33) |
| Mid:< 177.898 | 0.67 (0.26, 1.68) | | 0.85 (0.49, 1.47) |
| High:>= 177.898 | 1.41 (0.58, 3.46) | | 1.13 (0.63, 2.03) |
| Unspecified |  | |  |
| Low:< 5.127 | 1.02 (0.84, 1.24) | | 1.00 (0.86, 1.17) |
| Mid:< 177.898 | 1.11 (0.93, 1.33) | | 1.06 (0.92, 1.22) |
| High:>= 177.898 | 0.96 (0.79, 1.17) | | 1.02 (0.87, 1.18) |
| Glyphosate |  | |  |
| DLBCL |  | |  |
| Low:<= 2.4 | 1.03 (0.93, 1.14) | | 1.04 (0.95, 1.13) |
| Mid:<= 50.6 | 0.94 (0.85, 1.04) | | 0.95 (0.87, 1.04) |
| High:> 50.6 | 0.99 (0.89, 1.10) | | 1.00 (0.92, 1.10) |
| Follicular lymphoma |  | |  |
| Low:<= 2.4 | 1.15 (0.88, 1.51) | | 1.16 (0.94, 1.43) |
| Mid:<= 50.6 | 0.86 (0.65, 1.15) | | 1.01 (0.82, 1.25) |
| High:> 50.6 | 1.04 (0.78, 1.37) | | 1.08 (0.87, 1.33) |
| Burkitt lymphoma |  | |  |
| Low:<= 2.4 | 0.82 (0.45, 1.49) | | 0.83 (0.47, 1.44) |
| Mid:<= 50.6 | 1.72 (1.03, 2.88) | | 1.86 (1.17, 2.97) |
| High:> 50.6 | 0.91 (0.52, 1.60) | | 1.01 (0.6, 1.72) |
| Mantle cell lymphoma |  | |  |
| Low:<= 2.4 | 0.85 (0.63, 1.15) | | 0.88 (0.68, 1.14) |
| Mid:<= 50.6 | 1.13 (0.83, 1.54) | | 1.13 (0.87, 1.47) |
| High:> 50.6 | 0.89 (0.64, 1.24) | | 0.87 (0.65, 1.16) |
| Marginal zone lymphoma |  | |  |
| Low:<= 2.4 | 0.86 (0.51, 1.47) | | 1.04 (0.77, 1.42) |
| Mid:<= 50.6 | 1.07 (0.65, 1.78) | | 1.01 (0.72, 1.41) |
| High:> 50.6 | 1.03 (0.59, 1.78) | | 0.94 (0.65, 1.34) |
| Small lymphocytic lymphoma |  | |  |
| Low:<= 2.4 | 1.29 (0.61, 2.72) | | 0.99 (0.65, 1.50) |
| Mid:<= 50.6 | 1.22 (0.59, 2.53) | | 0.96 (0.65, 1.42) |
| High:> 50.6 | 1.25 (0.61, 2.55) | | 0.95 (0.64, 1.43) |
| Other B-cell lymphoma |  | |  |
| Low:<= 2.4 | 0.77 (0.27, 2.26) | | 0.76 (0.39, 1.49) |
| Mid:<= 50.6 | 0.97 (0.42, 2.21) | | 1.29 (0.78, 2.12) |
| High:> 50.6 | 1.09 (0.49, 2.41) | | 1.04 (0.60, 1.82) |
| T/NK-cell lymphoid neoplasms |  | |  |
| Low:<= 2.4 | 1.00 (0.79, 1.26) | | 1.05 (0.86, 1.29) |
| Mid:<= 50.6 | 1.05 (0.84, 1.32) | | 1.07 (0.88, 1.30) |
| High:> 50.6 | 0.99 (0.79, 1.25) | | 0.97 (0.79, 1.19) |
| Lymphoblastic lymphoma |  | |  |
| Low:<= 2.4 | 0.93 (0.30, 2.88) | | 1.21 (0.61, 2.43) |
| Mid:<= 50.6 | 1.74 (0.75, 4.05) | | 1.21 (0.69, 2.12) |
| High:> 50.6 | 0.88 (0.30, 2.55) | | 1.16 (0.62, 2.17) |
| Unspecified |  | |  |
| Low:<= 2.4 | 0.93 (0.75, 1.16) | | 0.97 (0.82, 1.14) |
| Mid:<= 50.6 | 1.06 (0.85, 1.31) | | 1.10 (0.93, 1.30) |
| High:> 50.6 | 0.94 (0.77, 1.15) | | 1.04 (0.89, 1.22) |
| Organophosphorus |  | |  |
| DLBCL |  | |  |
| Low:<= 1.3 | 0.87 (0.77, 0.97) | | 0.90 (0.82, 0.99) |
| Mid:<= 26.3 | 0.96 (0.86, 1.07) | | 0.97 (0.88, 1.07) |
| High:> 26.3 | 0.96 (0.85, 1.08) | | 1.00 (0.90, 1.10) |
| Follicular lymphoma |  | |  |
| Low:<= 1.3 | 0.80 (0.57, 1.12) | | 0.80 (0.62, 1.04) |
| Mid:<= 26.3 | 0.92 (0.67, 1.24) | | 1.08 (0.86, 1.35) |
| High:> 26.3 | 0.95 (0.69, 1.31) | | 0.95 (0.74, 1.21) |
| Burkitt lymphoma |  | |  |
| Low:<= 1.3 | 1.68 (1.00, 2.81) | | 1.61 (1.00, 2.60) |
| Mid:<= 26.3 | 0.92 (0.46, 1.81) | | 1.18 (0.67, 2.09) |
| High:> 26.3 | 0.97 (0.51, 1.85) | | 0.97 (0.52, 1.80) |
| Mantle cell lymphoma |  | |  |
| Low:<= 1.3 | 0.98 (0.69, 1.38) | | 0.98 (0.73, 1.32) |
| Mid:<= 26.3 | 1.03 (0.73, 1.44) | | 0.97 (0.71, 1.30) |
| High:> 26.3 | 0.80 (0.55, 1.15) | | 0.81 (0.60, 1.11) |
| Marginal zone lymphoma |  | |  |
| Low:<= 1.3 | 0.61 (0.32, 1.17) | | 1.09 (0.78, 1.51) |
| Mid:<= 26.3 | 0.66 (0.34, 1.26) | | 0.77 (0.52, 1.15) |
| High:> 26.3 | 0.99 (0.55, 1.79) | | 1.02 (0.70, 1.49) |
| Small lymphocytic lymphoma |  | |  |
| Low:<= 1.3 | 1.71 (0.84, 3.50) | | 1.46 (0.99, 2.16) |
| Mid:<= 26.3 | 0.55 (0.19, 1.55) | | 0.64 (0.38, 1.08) |
| High:> 26.3 | 0.77 (0.35, 1.71) | | 0.91 (0.60, 1.37) |
| Other B-cell lymphoma |  | |  |
| Low:<= 1.3 | 0.65 (0.20, 2.13) | | 0.77 (0.40, 1.49) |
| Mid:<= 26.3 | 0.84 (0.35, 1.98) | | 0.90 (0.51, 1.60) |
| High:> 26.3 | 1.32 (0.55, 3.20) | | 1.23 (0.65, 2.31) |
| T/NK-cell lymphoid neoplasms |  | |  |
| Low:<= 1.3 | 0.99 (0.76, 1.27) | | 1.06 (0.85, 1.32) |
| Mid:<= 26.3 | 1.06 (0.83, 1.34) | | 0.97 (0.78, 1.20) |
| High:> 26.3 | 0.91 (0.69, 1.19) | | 0.94 (0.74, 1.19) |
| Lymphoblastic lymphoma |  | |  |
| Low:<= 1.3 | 0.50 (0.12, 2.15) | | 0.88 (0.42, 1.84) |
| Mid:<= 26.3 | 0.77 (0.22, 2.71) | | 1.07 (0.52, 2.24) |
| High:> 26.3 | 1.38 (0.51, 3.75) | | 1.25 (0.65, 2.40) |
| Unspecified |  | |  |
| Low:<= 1.3 | 1.11 (0.88, 1.4) | | 1.04 (0.86, 1.25) |
| Mid:<= 26.3 | 1.20 (0.96, 1.50) | | 1.09 (0.92, 1.30) |
| High:> 26.3 | 0.92 (0.73, 1.17) | | 0.98 (0.82, 1.17) |
| Carbamate |  | |  |
| DLBCL |  | |  |
| Low:<= 0.2 | 1.05 (0.89, 1.23) | | 1.00 (0.86, 1.15) |
| Mid:<= 3.9 | 0.75 (0.62, 0.91) | | 0.82 (0.70, 0.96) |
| High:> 3.9 | 0.91 (0.76, 1.08) | | 1.01 (0.88, 1.17) |
| Follicular lymphoma |  | |  |
| Low:<= 0.2 | 0.89 (0.55, 1.42) | | 0.95 (0.67, 1.36) |
| Mid:<= 3.9 | 0.85 (0.53, 1.36) | | 0.85 (0.58, 1.23) |
| High:> 3.9 | 1.06 (0.66, 1.71) | | 1.09 (0.76, 1.57) |
| Burkitt lymphoma |  | |  |
| Low:<= 0.2 | 1.48 (0.57, 3.88) | | 2.27 (1.03, 4.97) |
| Mid:<= 3.9 | 0.65 (0.24, 1.76) | | 0.58 (0.22, 1.54) |
| High:> 3.9 | 1.59 (0.56, 4.46) | | 1.79 (0.71, 4.53) |
| Mantle cell lymphoma |  | |  |
| Low:<= 0.2 | 0.90 (0.56, 1.43) | | 0.94 (0.63, 1.39) |
| Mid:<= 3.9 | 0.98 (0.53, 1.80) | | 0.79 (0.44, 1.42) |
| High:> 3.9 | 0.86 (0.49, 1.52) | | 0.85 (0.52, 1.39) |
| Marginal zone lymphoma |  | |  |
| Low:<= 0.2 | 0.95 (0.46, 1.95) | | 0.94 (0.58, 1.53) |
| Mid:<= 3.9 | 1.39 (0.64, 3.02) | | 1.23 (0.74, 2.04) |
| High:> 3.9 | 1.12 (0.48, 2.59) | | 0.81 (0.46, 1.43) |
| Small lymphocytic lymphoma |  | |  |
| Low:<= 0.2 | 1.20 (0.42, 3.47) | | 0.82 (0.44, 1.56) |
| Mid:<= 3.9 | 1.07 (0.25, 4.57) | | 0.84 (0.34, 2.07) |
| High:> 3.9 | 0.56 (0.17, 1.91) | | 1.11 (0.64, 1.94) |
| Other B-cell lymphoma |  | |  |
| Low:<= 0.2 |  | | 1.12 (0.45, 2.77) |
| Mid:<= 3.9 | 1.29 (0.30, 5.47) | | 1.45 (0.58, 3.62) |
| High:> 3.9 | 0.59 (0.14, 2.56) | | 0.63 (0.22, 1.76) |
| T/NK-cell lymphoid neoplasms |  | |  |
| Low:<= 0.2 | 0.71 (0.47, 1.05) | | 0.71 (0.50, 1.00) |
| Mid:<= 3.9 | 0.84 (0.57, 1.22) | | 0.87 (0.63, 1.21) |
| High:> 3.9 | 0.99 (0.68, 1.45) | | 1.06 (0.76, 1.46) |
| Lymphoblastic lymphoma |  | |  |
| Low:<= 0.2 | 1.37 (0.18, 10.57) | | 0.77 (0.18, 3.23) |
| Mid:<= 3.9 | 6.77 (2.20, 20.80) | | 2.13 (0.76, 5.99) |
| High:> 3.9 | 4.19 (0.96, 18.26) | | 1.85 (0.61, 5.58) |
| Unspecified |  | |  |
| Low:<= 0.2 | 0.98 (0.69, 1.38) | | 0.95 (0.73, 1.24) |
| Mid:<= 3.9 | 0.99 (0.70, 1.41) | | 1.06 (0.82, 1.38) |
| High:> 3.9 | 1.09 (0.78, 1.51) | | 0.92 (0.70, 1.22) |
| Phenoxyherbicide |  | |  |
| DLBCL |  | |  |
| Low:<= 2.2 | 1.00 (0.86, 1.16) | | 1.02 (0.90, 1.16) |
| Mid:<= 23.1 | 1.06 (0.91, 1.24) | | 1.09 (0.96, 1.24) |
| High:> 23.1 | 1.09 (0.93, 1.27) | | 1.12 (0.98, 1.28) |
| Follicular lymphoma |  | |  |
| Low:<= 2.2 | 1.09 (0.73, 1.63) | | 1.09 (0.80, 1.48) |
| Mid:<= 23.1 | 0.93 (0.62, 1.39) | | 0.94 (0.69, 1.28) |
| High:> 23.1 | 0.98 (0.65, 1.48) | | 1.06 (0.77, 1.44) |
| Burkitt lymphoma |  | |  |
| Low:<= 2.2 | 0.69 (0.27, 1.76) | | 0.95 (0.44, 2.01) |
| Mid:<= 23.1 | 0.87 (0.39, 1.93) | | 0.90 (0.42, 1.90) |
| High:> 23.1 | 1.55 (0.70, 3.47) | | 1.67 (0.78, 3.56) |
| Mantle cell lymphoma |  | |  |
| Low:<= 2.2 | 0.51 (0.31, 0.85) | | 0.63 (0.43, 0.94) |
| Mid:<= 23.1 | 0.85 (0.50, 1.45) | | 0.77 (0.47, 1.24) |
| High:> 23.1 | 0.77 (0.48, 1.24) | | 0.86 (0.58, 1.27) |
| Marginal zone lymphoma |  | |  |
| Low:<= 2.2 | 1.07 (0.49, 2.31) | | 0.92 (0.55, 1.53) |
| Mid:<= 23.1 | 0.27 (0.07, 1.10) | | 0.33 (0.15, 0.74) |
| High:> 23.1 | 1.51 (0.72, 3.14) | | 1.23 (0.75, 2.03) |
| Small lymphocytic lymphoma |  | |  |
| Low:<= 2.2 | 0.73 (0.22, 2.44) | | 0.86 (0.47, 1.57) |
| Mid:<= 23.1 | 1.11 (0.43, 2.91) | | 1.02 (0.58, 1.79) |
| High:> 23.1 | 1.35 (0.57, 3.24) | | 1.09 (0.65, 1.85) |
| Other B-cell lymphoma |  | |  |
| Low:<= 2.2 | 0.92 (0.28, 3.04) | | 0.84 (0.36, 1.94) |
| Mid:<= 23.1 | 1.51 (0.48, 4.72) | | 1.73 (0.82, 3.66) |
| High:> 23.1 | 1.15 (0.33, 4.06) | | 1.12 (0.44, 2.88) |
| T/NK-cell lymphoid neoplasms |  | |  |
| Low:<= 2.2 | 1.14 (0.83, 1.57) | | 1.13 (0.85, 1.50) |
| Mid:<= 23.1 | 0.92 (0.64, 1.34) | | 0.86 (0.61, 1.20) |
| High:> 23.1 | 0.80 (0.57, 1.14) | | 0.83 (0.61, 1.12) |
| Lymphoblastic lymphoma |  | |  |
| Low:<= 2.2 | 2.47 (0.89, 6.84) | | 2.28 (1.13, 4.60) |
| Mid:<= 23.1 | 0.72 (0.15, 3.38) | | 0.37 (0.09, 1.56) |
| High:> 23.1 | 1.09 (0.20, 5.99) | | 1.68 (0.63, 4.45) |
| Unspecified |  | |  |
| Low:<= 2.2 | 0.86 (0.61, 1.22) | | 1.12 (0.89, 1.42) |
| Mid:<= 23.1 | 0.96 (0.70, 1.31) | | 0.95 (0.74, 1.22) |
| High:> 23.1 | 0.91 (0.68, 1.22) | | 0.88 (0.70, 1.12) |
| 2,4-Dimethylamine salt |  | |  |
| DLBCL |  | |  |
| Low:<= 1.8 | 0.94 (0.80, 1.11) | | 0.97 (0.84, 1.11) |
| Mid:<= 18.9 | 1.08 (0.92, 1.27) | | 1.11 (0.97, 1.27) |
| High:> 18.9 | 1.07 (0.91, 1.25) | | 1.11 (0.97, 1.27) |
| Follicular lymphoma |  | |  |
| Low:<= 1.8 | 0.97 (0.63, 1.48) | | 1.00 (0.73, 1.38) |
| Mid:<= 18.9 | 1.13 (0.77, 1.67) | | 1.01 (0.74, 1.38) |
| High:> 18.9 | 1.01 (0.65, 1.56) | | 1.03 (0.74, 1.45) |
| Burkitt lymphoma |  | |  |
| Low:<= 1.8 | 0.79 (0.28, 2.24) | | 1.06 (0.45, 2.50) |
| Mid:<= 18.9 | 0.97 (0.45, 2.07) | | 1.06 (0.53, 2.13) |
| High:> 18.9 | 1.23 (0.53, 2.86) | | 1.34 (0.61, 2.97) |
| Mantle cell lymphoma |  | |  |
| Low:<= 1.8 | 0.47 (0.27, 0.82) | | 0.62 (0.41, 0.95) |
| Mid:<= 18.9 | 1.05 (0.62, 1.78) | | 1.00 (0.63, 1.59) |
| High:> 18.9 | 0.82 (0.49, 1.35) | | 0.83 (0.54, 1.27) |
| Marginal zone lymphoma |  | |  |
| Low:<= 1.8 | 0.99 (0.43, 2.26) | | 0.88 (0.51, 1.52) |
| Mid:<= 18.9 | 0.29 (0.07, 1.17) | | 0.42 (0.20, 0.89) |
| High:> 18.9 | 1.59 (0.76, 3.33) | | 1.29 (0.78, 2.12) |
| Small lymphocytic lymphoma |  | |  |
| Low:<= 1.8 | 0.74 (0.18, 3.10) | | 0.93 (0.47, 1.83) |
| Mid:<= 18.9 | 1.46 (0.60, 3.57) | | 1.14 (0.65, 1.99) |
| High:> 18.9 | 1.14 (0.46, 2.84) | | 1.00 (0.59, 1.70) |
| Other B-cell lymphoma |  | |  |
| Low:<= 1.8 | 1.18 (0.41, 3.38) | | 0.85 (0.37, 1.98) |
| Mid:<= 18.9 | 1.64 (0.47, 5.80) | | 2.12 (0.97, 4.61) |
| High:> 18.9 | 0.77 (0.17, 3.43) | | 0.90 (0.32, 2.53) |
| T/NK-cell lymphoid neoplasms |  | |  |
| Low:<= 1.8 | 1.18 (0.84, 1.65) | | 1.13 (0.83, 1.52) |
| Mid:<= 18.9 | 0.90 (0.62, 1.30) | | 0.90 (0.65, 1.24) |
| High:> 18.9 | 0.78 (0.55, 1.11) | | 0.80 (0.59, 1.09) |
| Lymphoblastic lymphoma |  | |  |
| Low:<= 1.8 | 3.04 (1.10, 8.46) | | 2.00 (0.92, 4.32) |
| Mid:<= 18.9 | 0.96 (0.20, 4.76) | | 0.49 (0.11, 2.10) |
| High:> 18.9 | 0.84 (0.17, 4.07) | | 0.97 (0.37, 2.51) |
| Unspecified |  | |  |
| Low:<= 1.8 | 0.73 (0.50, 1.07) | | 1.05 (0.81, 1.35) |
| Mid:<= 18.9 | 0.97 (0.70, 1.34) | | 1.02 (0.80, 1.31) |
| High:> 18.9 | 0.88 (0.64, 1.19) | | 0.84 (0.65, 1.08) |
| **Cumulative pesticide exposure was measured as pounds of pesticide applied per acre/month within 2000 meters from residence at diagnosis, between 10 years prior up to 1 year after NHL diagnosis* | | | |
| *** Each pesticide is stratified by low, mid and high pesticide exposure levels based on the tertile distribution* | | | |
| ****Models were adjusted for gender, race/ethnicity, age at diagnosis, health insurance status, neighborhood socioeconomic status, rural or urban medical service study area and were stratified by stage at diagnosis and modality of initial therapy which included chemotherapy or radiation* | | | |
| *****Total refers to glyphosate, organophosphorus, carbamate, phenoxyherbicide and 2,4-dimethylamine salt combined* | | | |
| *NHL - non-Hodgkin lymphoma; DLBCL - diffuse large B-cell lymphoma* | |  | |
